# Theoretical and Experimental Studies on Quantitative Analysis of Impact-Penetration Properties of BFRP Laminates

**DOI:** 10.3390/ma19214664

**Published:** 2026-11-02

**Authors:** Zebing Fan, Dongyang Cui, Yucheng Zhou, Hongjie Zhu

**Affiliations:** 1 AECC Sichuan Gas Turbine Establishment, Mianyang 621000, China; fanzebing198@gmail.com; 2 School of Civil Engineering and Architecture, Southwest University of Science and Technology, Mianyang 621010, China; bankaiwen494@gmail.com (D.C.); zhouyucheng@mails.swust.edu.cn (Y.Z.)

**Keywords:** BFRP laminates, impact-penetration properties

## Abstract

Basalt fiber-reinforced polymer (BFRP) laminates have attracted increasing attention in engineering applications owing to their favorable mechanical properties. However, the influence of fiber content and stacking sequence on their low-velocity impact response and damage evolution remains insufficiently understood. To address this gap, nine groups of BFRP laminates with different fiber contents and stacking sequences were subjected to drop-weight impact tests. Contact force–time histories, displacement responses, and failure characteristics were analyzed to evaluate their impact performance. An analytical model based on energy conservation was also developed to describe the relationship between contact force and penetration depth, accounting for energy dissipation associated with laminate bending, interlaminar delamination, and material failure, as well as the effects of laminate stiffness and penetration resistance. The results show that increasing fiber content leads to more pronounced post-peak force fluctuations, accompanied by progressive damage development under the investigated conditions. Among the three stacking configurations, the [±30] laminates exhibited the smallest damage area and a comparatively favorable impact response, whereas the [0/90] laminates suffered more severe damage. The predicted force–penetration responses agreed reasonably well with the experimental results, indicating that the model captures the overall response trends under the investigated conditions. The proposed model therefore provides a simplified analytical approach for describing the low-velocity impact response of BFRP laminates.

## 1. Introduction

Basalt fiber-reinforced polymer (BFRP) composites have attracted increasing attention in aerospace, transportation, and lightweight structural applications because of their favorable mechanical properties, relatively low cost, and potential environmental benefits [1,2,3]. During service, however, BFRP components may be subjected to low-velocity impacts resulting from dropped objects, foreign-object strikes, or accidental tool contact. Such localized impacts can induce matrix cracking, interlaminar delamination, and fiber fracture, even when visible surface damage is limited. Under subsequent repeated or sustained loading, such damage may propagate and compromise structural performance. Previous studies have shown that impact-induced damage can significantly reduce the residual load-carrying capacity of fiber-reinforced composite structures [2]. Understanding the impact response and damage evolution of BFRP laminates is therefore essential for evaluating their structural performance and ensuring their safe application in engineering structures.

Considerable research has been conducted on the low-velocity impact behavior of fiber-reinforced polymer composites. Li et al. [1] combined impact tests with finite element analysis to investigate penetration damage in hybrid composite laminates and identified distinct failure mechanisms in the front and rear layers. Bunea et al. [3], Wang et al. [4], and Liu et al. [5] showed that impact energy is mainly dissipated through fiber fracture, matrix cracking, and interfacial delamination. Sharan et al. [6] investigated polyethylene self-reinforced composites and demonstrated that interfacial properties play an important role in determining the impact response. Liu et al. [7] developed a numerical framework incorporating failure criteria, cohesive-zone modeling, and stiffness degradation to simulate delamination evolution in stitched composite laminates. Wu et al. [8] further examined the low-velocity impact and post-impact compression behavior of hybrid braided laminates. Taken together, these studies suggest that the impact response of composite laminates is governed by the interaction of multiple damage mechanisms rather than a single failure mode.

Energy absorption and dissipation also provide important insights into the damage mechanisms of composite laminates under impact loading. Pushparaja et al. [9] reported that impact energy in CFRP laminates is dissipated mainly through matrix deformation, delamination, and fiber failure. Nisini et al. [10] investigated hybrid composite laminates and showed that stacking configuration can substantially alter the distribution of absorbed energy. Zobeiry et al. [11] developed an energy-based approach for damage characterization, while Velumayil et al. [12] demonstrated that the stacking sequence can significantly affect the mechanical and impact performance of basalt-fiber-containing composites. Jiwei et al. [13] also investigated the relationship between impact-induced damage and subsequent fatigue behavior in thermoplastic composites. Collectively, these studies indicate that energy-based descriptions are useful for linking macroscopic impact response to the underlying damage processes.

In parallel with experimental studies, various analytical and numerical approaches have been proposed to predict the impact response of composite laminates. Existing models include progressive-damage finite element formulations, cohesive-zone approaches, strain-rate-dependent constitutive models, and data-driven parameter-identification methods [14,15,16,17,18,19,20,21]. The influence of laminate thickness, ply orientation, stacking sequence, and interfacial properties has also been extensively investigated [17,22,23,24]. These approaches have contributed to improved predictions of impact-induced damage, particularly in CFRP and GFRP laminates. Nevertheless, the impact response of BFRP laminates remains comparatively less well characterized. Differences in fiber–matrix interaction, interfacial behavior, and anisotropic mechanical properties may lead to impact responses that differ from those of conventional CFRP and GFRP systems. Consequently, direct application of models developed for other composite systems may not always provide satisfactory predictions for BFRP laminates.

In addition, many existing studies rely on detailed numerical simulations that require numerous material parameters and considerable computational effort. For engineering applications, a simplified analytical model capable of quantitatively describing the relationship between impact force and penetration response would therefore be valuable. In particular, such a model should account for the major energy-dissipation mechanisms associated with laminate deformation, interfacial delamination, and material failure while retaining a relatively simple formulation.

To address these limitations, this study investigates the low-velocity impact behavior of BFRP laminates with different fiber contents and stacking sequences through experimental testing and theoretical analysis. Nine groups of drop-weight impact tests were conducted to characterize the contact force response, penetration and deformation behavior, and damage characteristics of the laminates. Based on energy conservation, an analytical model was developed to predict the contact force–penetration depth relationship by accounting for bending deformation energy, interlaminar delamination energy, and energy consumed by material failure. The model was validated against the experimental results, and the influences of fiber content, stacking sequence, laminate stiffness, and selected energy-dissipation parameters were further examined. The proposed approach is intended to provide a simplified theoretical framework for evaluating the low-velocity impact response of BFRP laminates.

## 2. Experimental Program

### 2.1. Specimen Preparation

**Figure 1:**
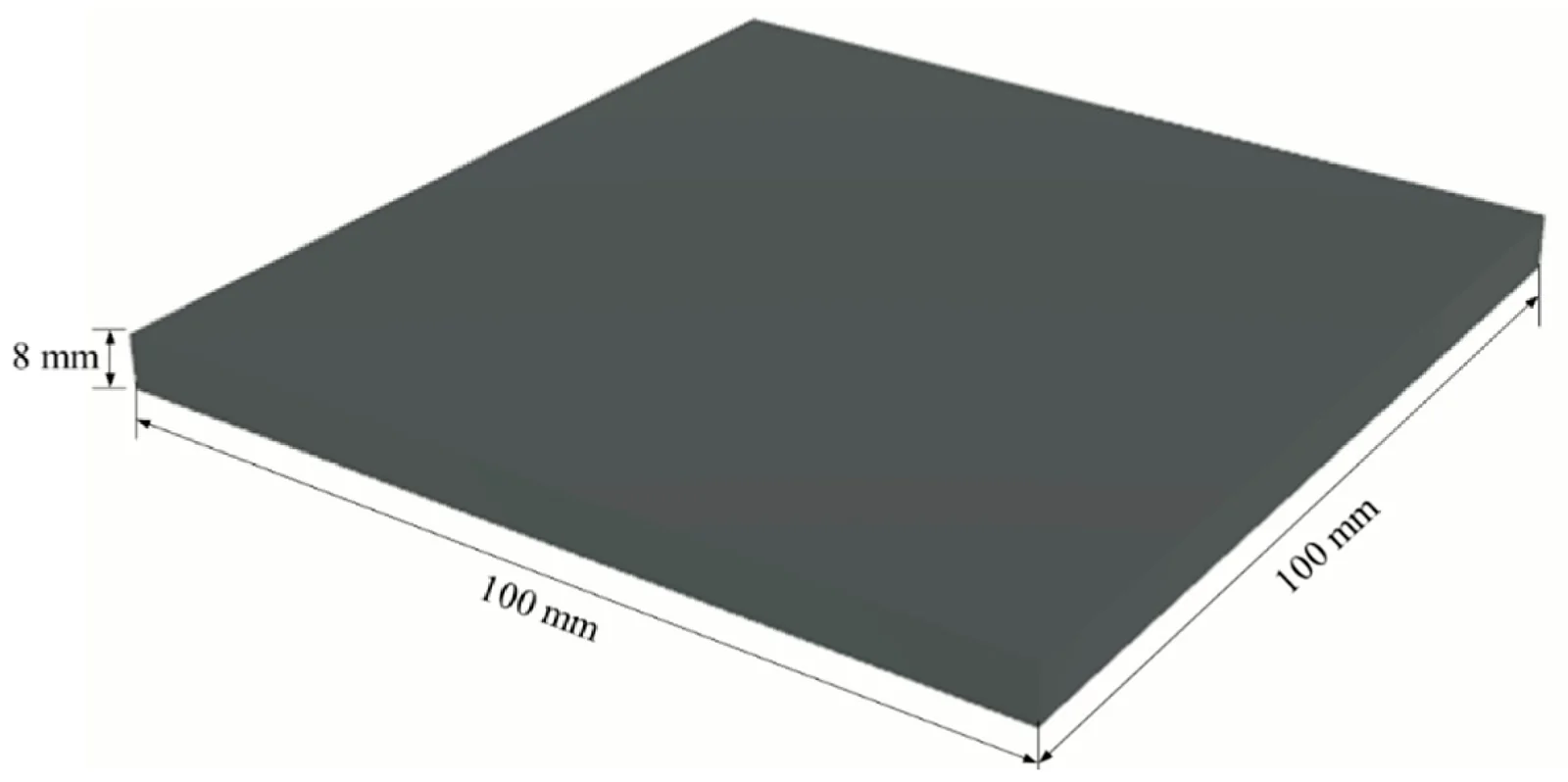
Sample diagram.

The BFRP laminates were fabricated by hot pressing 12 plies of BWR300-100 unidirectional basalt fiber fabric. The basic mechanical properties of the basalt fiber fabric and the consolidated BFRP laminates are summarized in Table 1. The tensile breaking forces per unit width of the basalt fiber fabric in the warp and weft directions were 1920 and 1959 N/mm, respectively, whereas the consolidated BFRP laminates exhibited flexural and compressive strengths of 372 and 220 MPa, respectively. During specimen fabrication, E-51 epoxy resin and bisphenol A dicyanate (BADCy) were mixed at a mass ratio of 55:45 to prepare the resin system. Acetone was added at a dosage of 25 g per 100 g of resin mixture, followed by heating and stirring at 90 °C until a homogeneous mixture was obtained. The resin solution was uniformly applied to the basalt fiber fabric, which was then repeatedly rolled to promote impregnation. The impregnated fabric was sealed in a vacuum bag and evacuated for 1 h. The prepregs were then pre-cured at 140 °C for 12 min, arranged according to the prescribed stacking sequences, and hot-pressed at 160 °C under 1.0 MPa for 4 h. Following natural cooling to room temperature, the laminates were cut into 100 mm × 100 mm specimens, as shown in Figure 1.

The mechanical-property values reported in Table 1 were obtained from independent experimental tests. The tensile breaking forces of the basalt fiber fabric in the warp and weft directions were determined using a strip tensile test method adapted from GB/T 7689.5-2001 [25]. The flexural strength of the consolidated BFRP laminates was determined by three-point bending according to GB/T 1449-2005 [26], while their in-plane compressive strength was evaluated according to ISO 14126:1999 [27]. The coefficients of variation of the four corresponding properties were 3.7%, 2.4%, 2.8%, and 1.9%, respectively.

The nominal dimensions of the specimens were 100 mm × 100 mm × 8.0 mm. Before impact testing, the thickness of each specimen was measured at three independent locations, and the arithmetic mean was calculated. The mean thicknesses of the 27 specimens are reported in Table 2, ranging from 7.96 to 8.04 mm, which falls within the specified tolerance of 8.00 ± 0.20 mm.

### 2.2. Test Procedure

**Figure 2:**
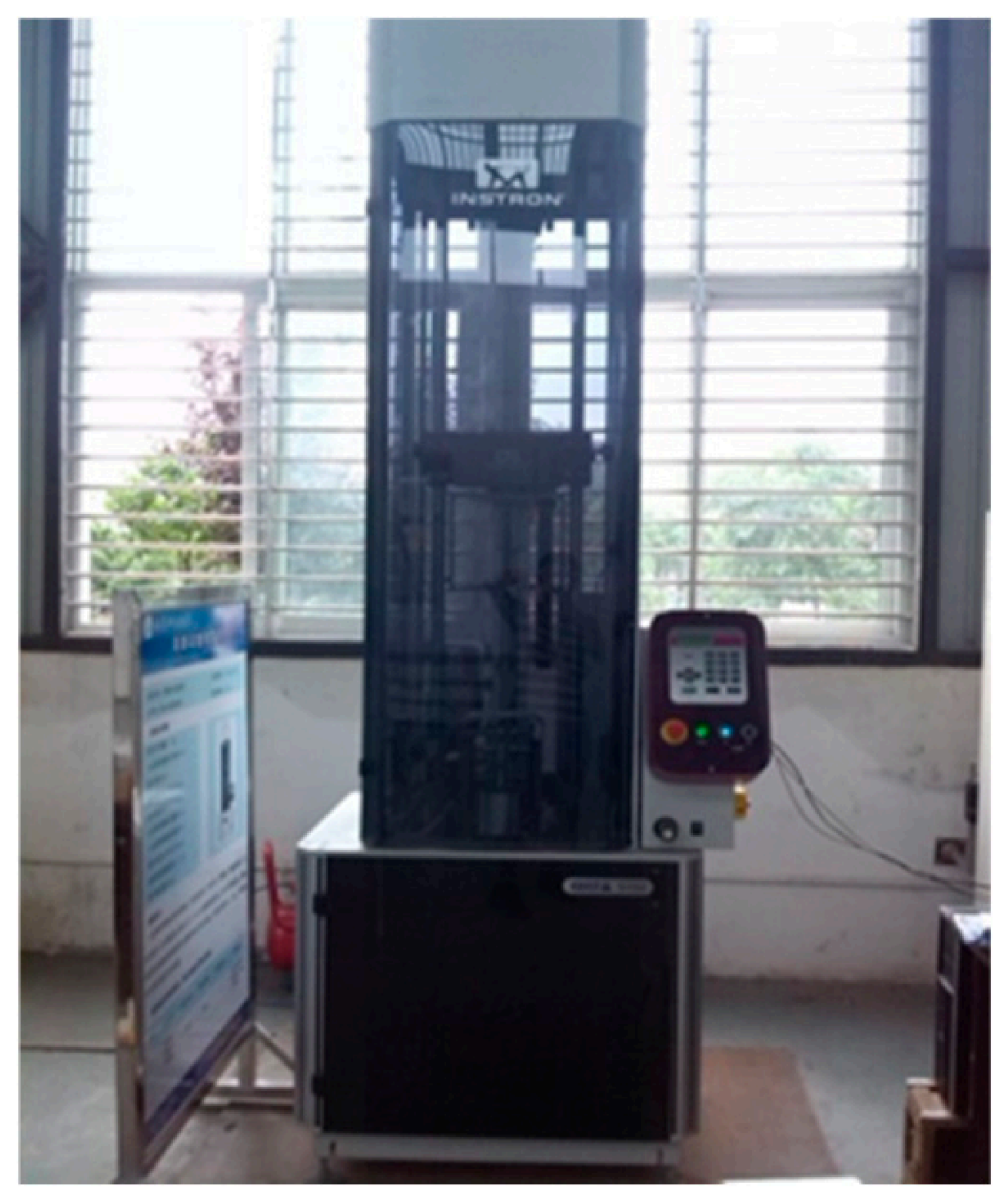
Drop hammer impact tester.

An INSTRON CEAST 9350 drop-weight impact system, consisting of a drop-weight tower and a DAS64K data acquisition system, was used to investigate the impact response of the BFRP laminates (Figure 2). A 20 mm diameter hemispherical metallic impactor with an effective moving assembly mass of 5.50 kg was employed. For a nominal incident energy of 30 J, the initial impact velocity was 3.303 m/s, and the contact force was sampled at 500 kHz. The apparatus was equipped with an anti-rebound mechanism to prevent unintended secondary impacts.

**Figure 3:**
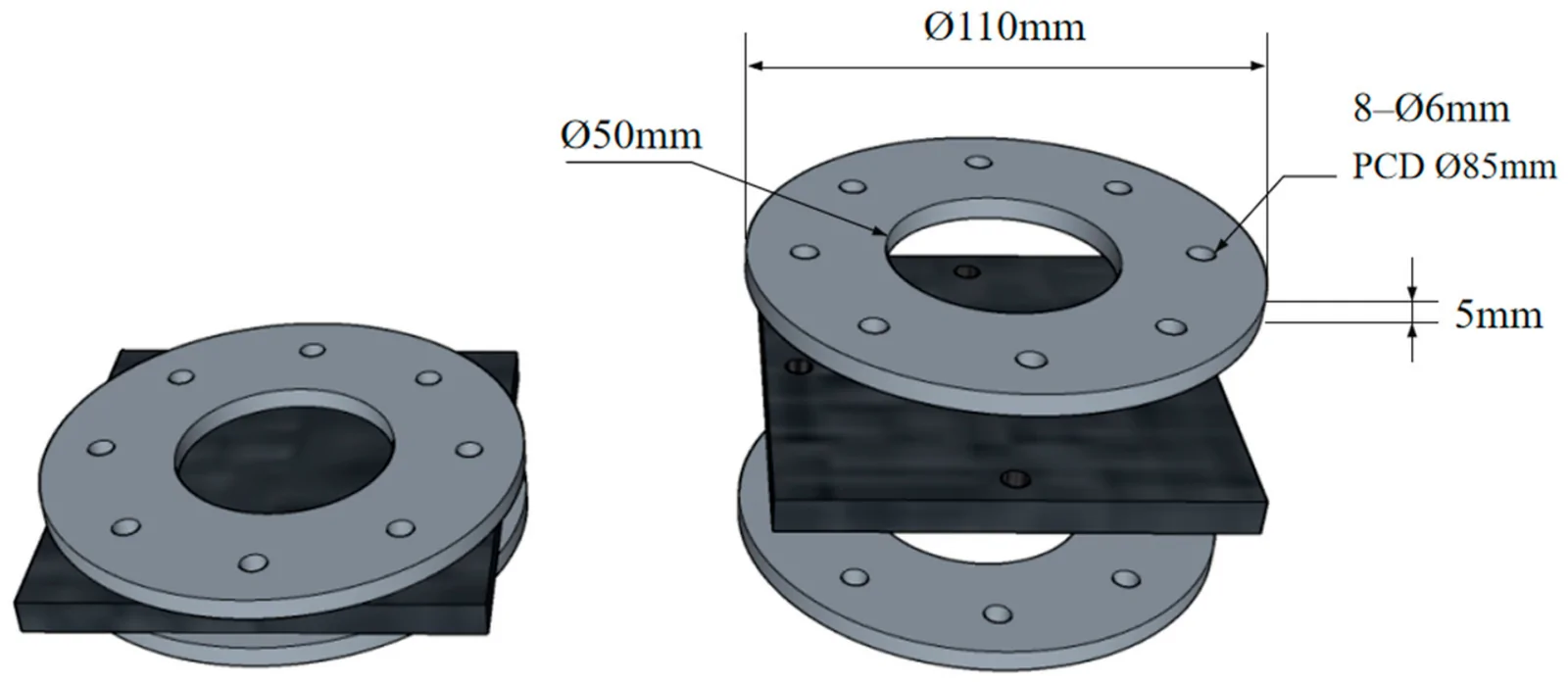
Schematic of the annular specimen clamping fixture.

The specimen fixture comprised upper and lower annular steel plates that securely clamped the BFRP laminate (Figure 3). Each plate had an outer diameter of 110 mm, a central opening of 50 mm in diameter, and a thickness of 5 mm. Eight bolt holes, each 6 mm in diameter, were uniformly distributed along a pitch circle diameter of 85 mm. The four peripheral holes in the specimens were pre-existing mounting holes rather than impact-induced perforations. The impactor was aligned with the central opening to apply the impact load to the center of the specimen. During testing, the data acquisition system recorded the time histories of the relevant impact-response parameters. The instrumented impact testing procedure was based on ISO 6603-2:2000 [28], with the nominal 8 mm specimen thickness treated as a deviation from the standard thickness range.

A common incident impact energy of 30 J was selected for the nine combinations of fiber mass fractions (40%, 60%, and 80%) and stacking sequences ([0/90], [0/45/90/−45], and [±30]). This energy level was chosen to induce observable impact damage and allow its progressive development to be examined under repeated loading while maintaining consistent impact conditions across all configurations. Higher incident energies (e.g., 40 or 50 J) could accelerate damage development and penetration, potentially limiting the investigation of intermediate damage stages; those higher energies were not directly compared in this program.

For the repeated-impact series, five successive impacts at 30 J were applied to the same central region of each 100 mm × 100 mm specimen. The specimens remained securely clamped between the upper and lower fixture plates, and the impactor was reset before each subsequent impact. The planned sequence ended after five impacts; through-thickness penetration was recorded as specimen failure. The peak contact force and rebound energy were recorded for each impact to characterize the evolution of the impact response during repeated loading.

The impact-test matrix comprised nine groups covering three fiber mass fractions and three stacking sequences, with three specimens per group (27 specimens in total). The prescribed specimen dimensions, stacking configurations, fiber mass fractions, and loading conditions are listed in Table 3.

## 3. Theoretical Model

### 3.1. Impact-Penetration Process Analysis

**Figure 4:**
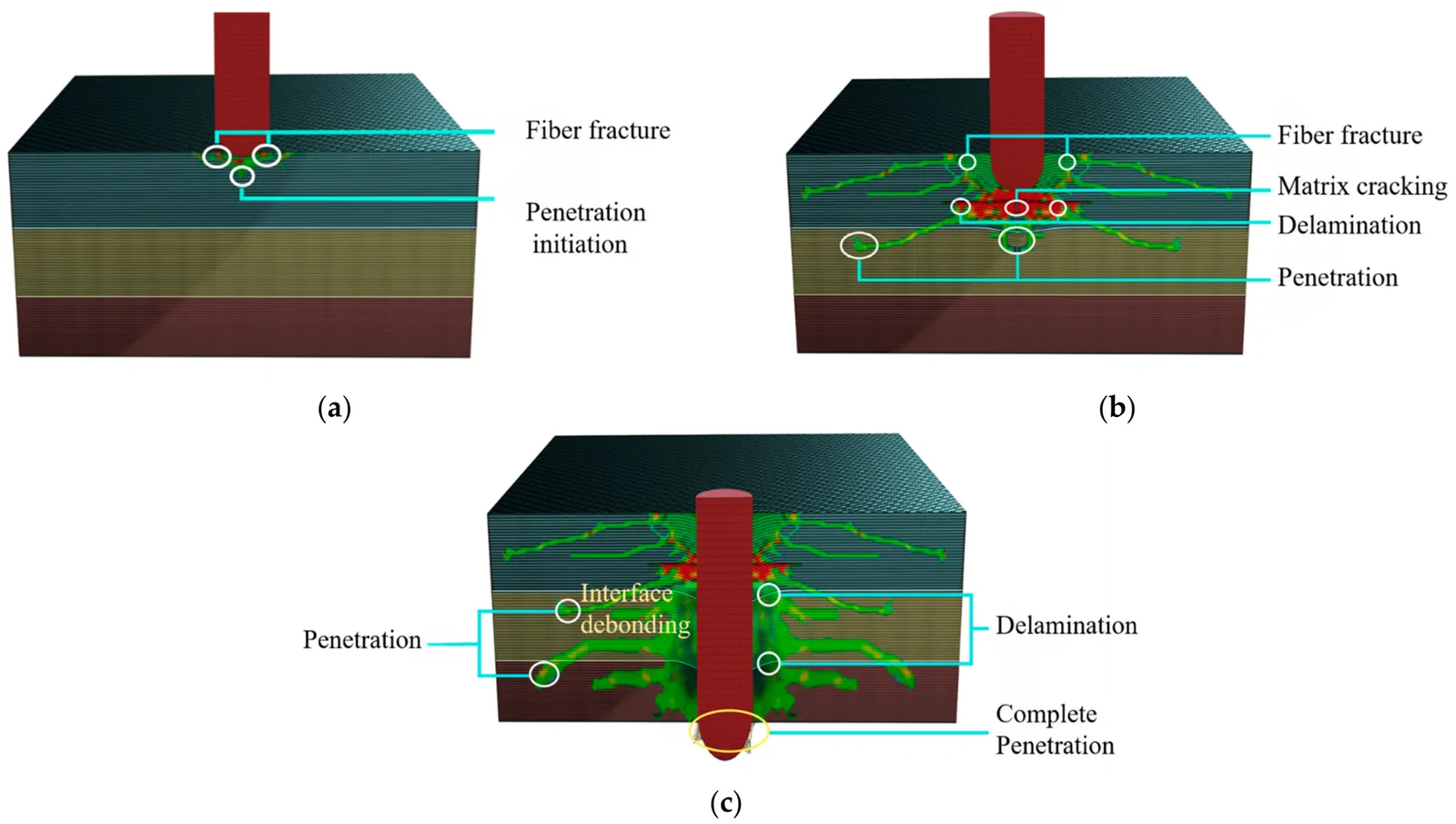
Schematic illustration of representative stages of low-velocity impact penetration of BFRP laminates: (**a**) initial contact and damage onset; (**b**) penetration initiation and progressive damage; and (**c**) complete penetration.

Figure 4 illustrates three representative stages of the low-velocity contact and penetration response of BFRP laminates. Panel (a) depicts initial contact and damage onset, including the localized fiber damage shown schematically, and therefore should not be interpreted as a purely elastic, undamaged stage. As penetration initiates and damage progresses in panel (b), matrix cracking, interfacial separation, and fiber damage may reduce local stiffness and contribute to a nonlinear contact-force response. Continued penetration eventually produces extensive fracture and complete perforation, as represented by panel (c). These illustrations depict representative response regimes rather than experimentally time-resolved transitions.

### 3.2. Theoretical Model of Impact Force During Penetration

During the entire penetration stage, the laminate underwent plastic deformation, fiber fracture, interface delamination, and matrix destruction. The contact stiffness decreases due to the increase in the cumulative damage to the laminate. At a given moment *i* during the impact process, energy dissipation occurred mainly in the form of plate bending deformation energy *E*_s_, interface delamination energy consumption *E*_d_, and the energy consumed by matrix and fiber failure *E*_c_. Therefore, by neglecting the energy losses due to the drop hammer and guide slide, fiber debris ejection, material damping, and energy dissipation from nonlinear vibrations, the law of conservation of energy yields:

(1) $${\dot{E}}_{s}+{\dot{E}}_{d}+{\dot{E}}_{c}=F\cdot \dot{\delta }$$

where ${\dot{E}}_{s}$, ${\dot{E}}_{d}$, and ${\dot{E}}_{c}$ represent the change rates of the bending deformation energy *E*_s_, interface delamination energy *E*_d_, and base layer failure energy *E*_c_ of the plate, respectively; $\dot{\delta }$ represents the change rate of the impact deflection; and *F* is the contact force.

The bending deformation energy *E*_s_ of the plate can be expressed as:

(2) $$E_{s}=\int_{0}^{\delta_{\max}}F_{1}d\delta$$

(3) $$F_{1}=k\delta$$

where *k* is the bending stiffness of the plate; *δ* is the deflection of the plate.

The stiffness *k* is calculated from the equivalent elastic modulus *E*_eq_ of the laminate:

(4) $$k=\frac{E_{\mathrm{eq}}A}{L}$$

The equivalent elastic modulus *E*_eq_ is calculated using the modified Reuss model [29]:

(5) $$E_{\mathrm{eq}}=\frac{E_{f}E_{m}}{V_{f}E_{m}+V_{m}E_{f}(1+\alpha \frac{tp}{r_{f}}\frac{E_{m}}{E_{p}})}$$

where *V*_f_ is the volume fraction of the fiber layer; *V*_m_ is the volume fraction of the matrix layer; *α* is the shape parameter of the laminate when it is impacted; *t*_p_ is the layer thickness of the fiber layer between the laminates; *r*_f_ is the radius of the fiber; *E*_p_ is the elastic modulus of the laminate interface layer; *E*_m_ is the elastic modulus of the matrix; and *E*_f_ is the elastic modulus of the fiber layer. Bringing Equations (3) and (4) into Equation (2) yields the following.

The 40%, 60%, and 80% fiber contents specified in the test matrix represent mass fractions. For the modified Reuss model, constituent volume fractions can be obtained using densities of 2.70 g/cm^3^ for basalt fibers and 1.25 g/cm^3^ for the fully cured resin matrix. Each constituent mass contribution is divided by its density to obtain a volume contribution; the individual volumes are then normalized by their total, assuming negligible void content. The corresponding fiber and matrix volume fractions are the constituent quantities required in Equation (5).

(6) $$E_{s,i}=\frac{k\delta_{i}^{2}}{2}$$

Delamination failure is mainly caused by shear stress. The energy *E*_d_ consumed by delamination of fiber composite materials can be expressed as:

(7) $$E_{d,i}=n_{i}A_{d}G$$

where *n_i_* is the number of delaminations between the sub-layers of the fiber composite laminate, which increases with the impact time, and the maximum number of layers is equal to the number of fiberboard layers. *G* is the energy consumed by the interface tearing per unit area to produce delamination. For the epoxy resin bonding layer, the value range of *G* is usually 500~1000 J/m^2^. *A*_d_ is the area of the delamination region. In the experiment, as the impact head penetrated the BFRP laminate vertically, the delamination area was approximately equal to the projected area of the circular impact head on the laminate.

The work done by the resistance is equal to the energy *E*_c_ consumed in the process of laminate crushing, which can be expressed as:

(8) $$E_{c,i}=\bar{\sigma }\cdot \frac{\pi R^{2}}{4}\cdot \delta_{i}$$

where $\bar{\sigma }$ is the equivalent resistance per unit area during the impact penetration of the impact head; *R* is the diameter of the projection of the contact surface between the spherical impact head and the laminate, *R* = *δ*^2^cos*θ*.

Wen [30] proposed that the average resistance of the fragments consists of two parts; one is the quasi-static resistance caused by the elastic-plastic deformation of the plate material, and the other is the dynamic resistance caused by the velocity effect. Therefore, the equivalent resistance during the penetration process can be expressed as:

(9) $$\bar{\sigma }=\sigma_{s}+\sigma_{d}$$

The quasi-static resistance $\sigma_{s}$ can be approximately expressed as:

(10) $$\sigma_{s}=\sigma_{c}$$

where $\sigma_{c}$ is the compressive strength of the laminate in the thickness direction.

The dynamic resistance $\sigma_{d,i}$ is considered to be a function of $\sqrt{\left(\frac{\rho_{t}}{\sigma_{e}}\right)}v_{i}$, and its expression is:

(11) $$\sigma_{d,i}=\beta_{0}\sqrt{\frac{\rho_{t}}{\sigma_{e}}}v_{i}\sigma_{c}$$

where $\sigma_{c}$ is the compressive strength in the quasi-static linear elastic range in the thickness direction of the laminate; $\rho_{t}$ is the density of the laminate; *β*_0_ = 3/4*ψ* is a constant related to the shape of the impact head (*ψ* is the ratio of the impact head curvature radius to the impact head diameter), and for a spherical impact head, *β*_0_ *=* 1.5. *v_i_* is the velocity of the hammer at the current moment, which decreases with the increase in the impact depth during the impact-penetration stage.

Substituting Equations (9)–(11) into Equation (8) yields Equation (12):

(12) $${\bar{\sigma }}_{i}=(1+\frac{3}{2}\sqrt{\frac{\rho_{t}}{\sigma_{e}}}v_{i})\sigma_{c}$$

Substituting Equation (12) into Equation (7) yields:

(13) $$E_{c,i}=(1+\frac{3}{2}\sqrt{\frac{\rho_{t}}{\sigma_{c}}}v_{i})\sigma_{c}\cdot \frac{\pi R^{2}}{4}\cdot \delta_{i}$$

Substituting Equations (5), (6) and (13) into Equation (1) yields:

(14) $$\frac{k\delta_{i}^{2}}{2}dt+n_{i}A_{d}Gdt+(1+\frac{3}{2}\sqrt{\frac{\rho_{t}}{\sigma_{c}}}v_{i})\sigma_{c}\cdot \frac{\pi R^{2}}{4}\cdot \delta_{i}dt=F_{i}\cdot \delta_{i}dt$$

where *n_i_* is the number of delaminations damaged by the impact of the falling hammer, and *δ_i_* is proportional to the relationship; *ρ_t_* is the basalt fiber plywood density, which takes 40~200kg/m^3^, and *A*_d_ is the projected area of the impact head in the plywood, *A*_d_ = *πR*^2^/4cos*θ. θ* is the angle between the impact head and the surface of the laminate. Since the impact experiments were conducted under vertical impact conditions, *θ* was taken as 90°, resulting in cos*θ* = 1 in the calculations [31]. Eventually, the integration of the left and right sides of the equation with respect to time is simplified to obtain the following equation:

(15) $$F=\frac{k\delta_{i}^{2}}{2}+nA_{d}G+(1+\frac{3}{2}\sqrt{\frac{\rho_{t}}{\sigma_{c}}}v_{i})\sigma_{c}\cdot \frac{\pi \delta_{i}^{4}}{4}$$

## 4. Results and Discussion

### 4.1. Impact Test Results

**Figure 5:**
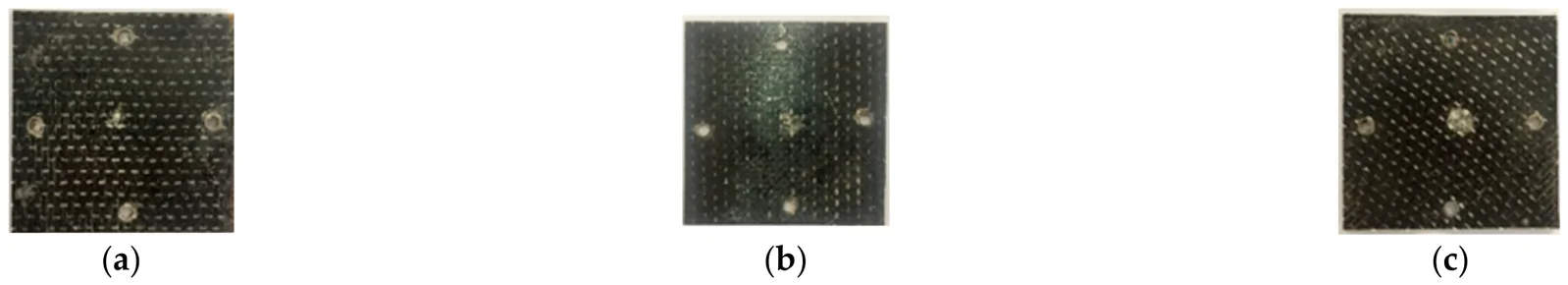
Representative post-impact surface morphologies of BFRP laminates with different fiber mass fractions: (**a**) 40%; (**b**) 60%; and (**c**) 80%.

**Figure 6:**
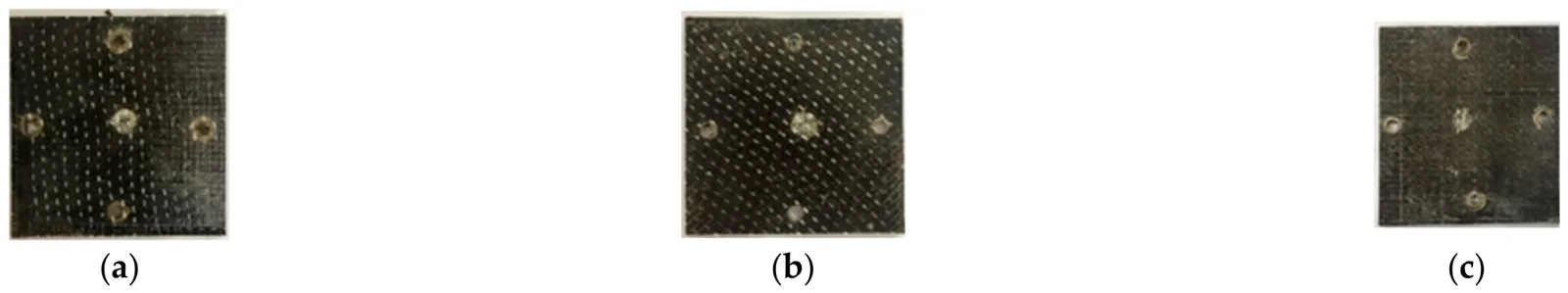
Representative post-impact surface morphologies of BFRP laminates with different stacking sequences: (**a**) [0/90]; (**b**) [0/45/90/−45]; and (**c**) [±30].

A total of 27 specimens, comprising nine groups, were subjected to drop-weight impact tests. The corresponding damage morphologies are presented in Figure 5 and Figure 6. For a given impact energy and stacking configuration, both the depth and diameter of the surface craters increased with fiber content, indicating progressively more severe impact-induced damage. Among the three stacking configurations, the [±30] laminates exhibited the smallest damaged areas, whereas the [0/90] laminates showed the most severe surface damage. These observations highlight the important role of stacking sequence in governing the impact response and damage characteristics of BFRP laminates.

**Figure 7:**
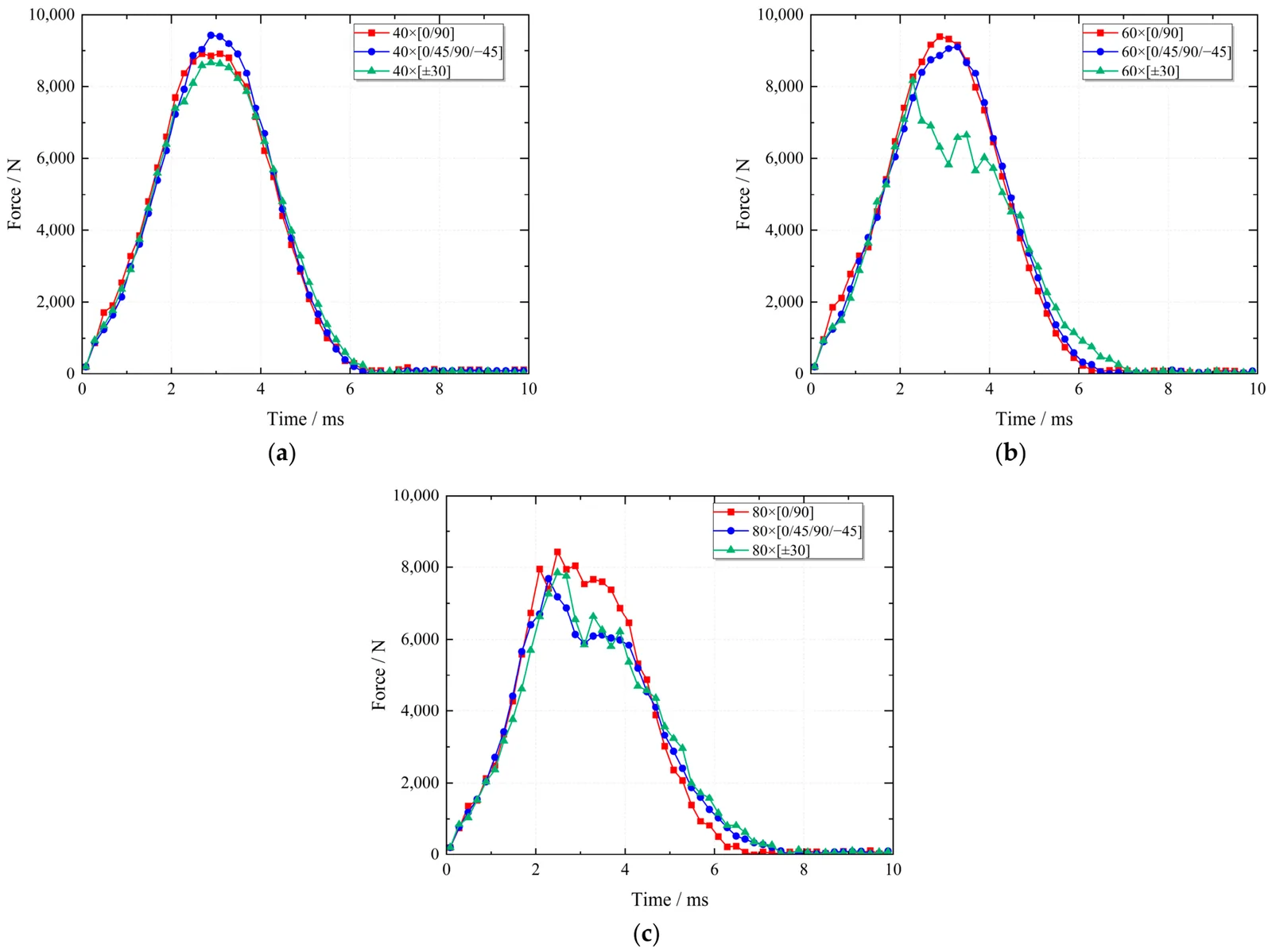
Contact force-time responses of BFRP laminates with different fiber mass fractions: (**a**) 40%; (**b**) 60%; and (**c**) 80%.

**Figure 8:**
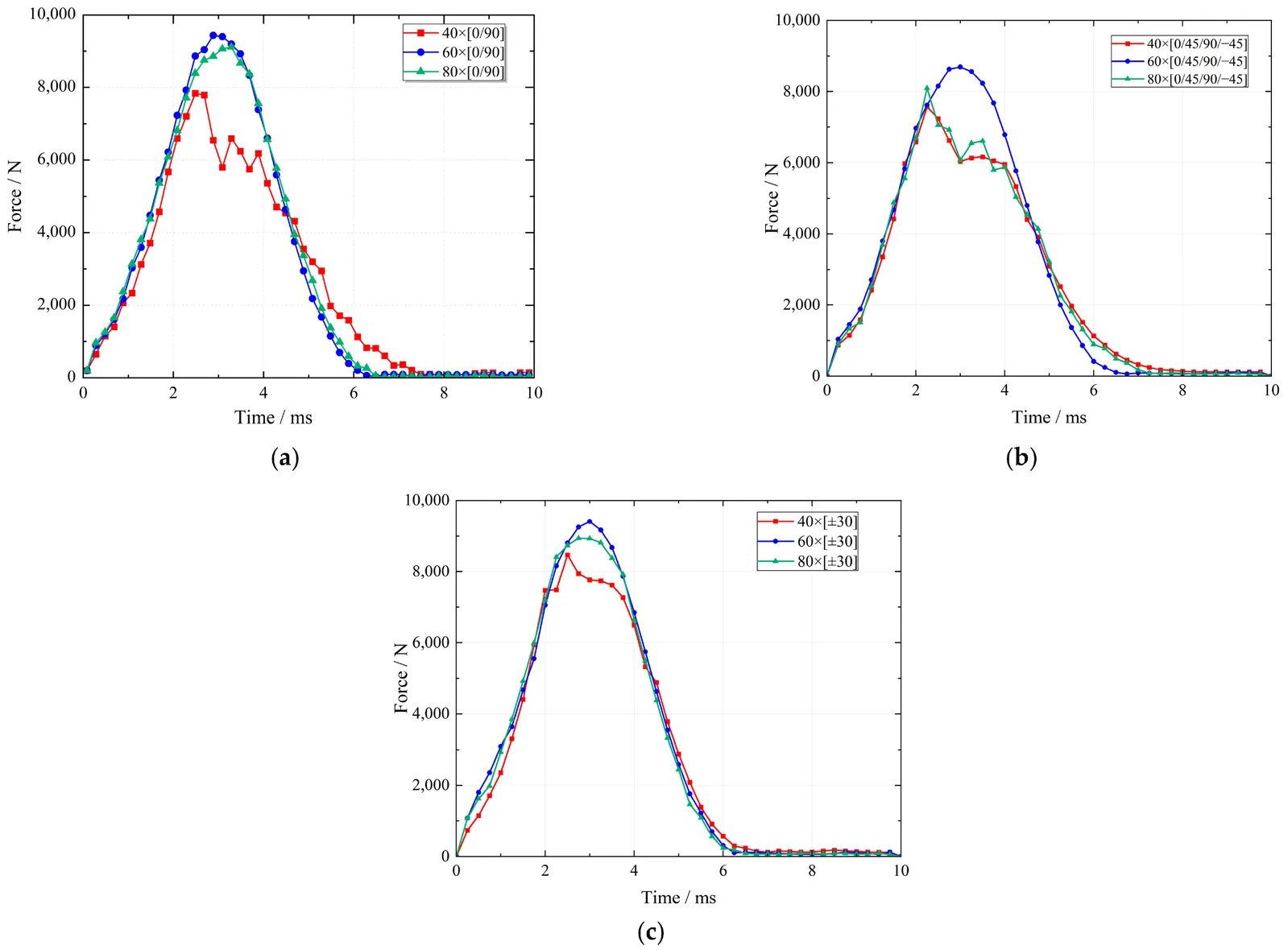
Contact force-time responses of BFRP laminates with different stacking sequences: (**a**) [0/90]; (**b**) [0/45/90/−45]; and (**c**) [±30].

The contact force–time histories obtained from the drop-weight impact tests are presented in Figure 7 and Figure 8. Overall, the specimens exhibited a typical single-peak response, characterized by a short initial loading stage followed by a rapid decrease in contact force after the peak. The contact duration was generally within the range of 6–8 ms. Figure 7 compares the representative impact responses of BFRP laminates with different fiber contents. At a fiber content of 40%, the contact force–time curve was relatively smooth and approximately parabolic, with only minor post-peak fluctuations, consistent with comparatively limited damage development. As the fiber content increased to 60% and 80%, the post-peak response became increasingly oscillatory, suggesting more pronounced damage evolution during impact. In particular, the specimens with 80% fiber content exhibited an earlier peak followed by a sharp reduction in contact force, indicating rapid damage development after peak loading. The pronounced post-peak fluctuations may reflect progressive internal damage, including delamination, matrix cracking, and fiber failure, together with a reduction in the effective stiffness of the laminate. These results indicate that fiber content influences both the magnitude and temporal evolution of the impact response.

Figure 8 compares the contact force–time responses of BFRP laminates with different stacking sequences at fiber contents of 40%, 60%, and 80%. At a fiber content of 40%, all three stacking configurations exhibited relatively smooth single-peak responses with only minor fluctuations, indicating a comparatively stable impact response. At 60% fiber content, the [0/90] laminate maintained a comparatively smooth single-peak response, with only slight fluctuations near the peak. In contrast, the [0/45/90/−45] laminate exhibited more pronounced fluctuations around the peak, suggesting more substantial damage evolution and stiffness degradation. When the fiber content increased to 80%, all three stacking configurations exhibited a rapid reduction in contact force after reaching the peak, followed by pronounced post-peak oscillations. This response indicates rapid damage development and progressive degradation of the effective stiffness after peak loading. The pronounced oscillations may be associated with the progressive development of multiple damage mechanisms, including matrix cracking, delamination, and fiber failure. Overall, the results suggest that the influence of stacking sequence on the impact response becomes more apparent at higher fiber contents.

**Figure 9:**
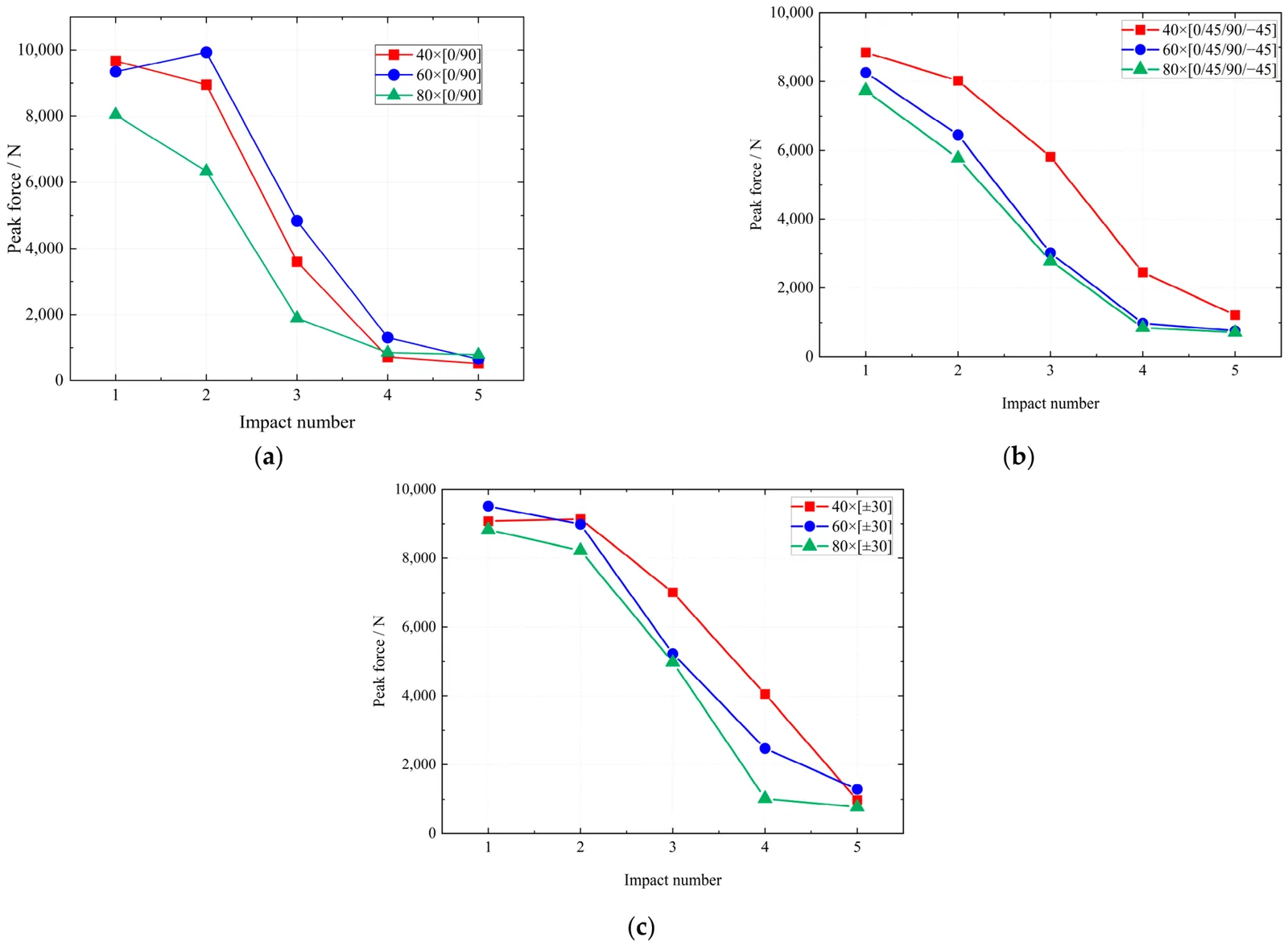
Peak contact forces of BFRP laminates with different fiber mass fractions and stacking sequences: (**a**) [0/90]; (**b**) [0/45/90/−45]; and (**c**) [±30].

Figure 9 compares the peak contact forces of BFRP laminates with different fiber contents and stacking sequences. Similar trends were observed for all three stacking configurations. As the fiber content increased from 40% to 80%, the peak contact force generally decreased, indicating a reduction in the peak load transmitted by the laminates under the tested impact conditions. This trend suggests that fiber content influences the dynamic load-carrying response of BFRP laminates; however, their overall impact resistance should also be assessed in terms of energy absorption and damage characteristics.

**Figure 10:**
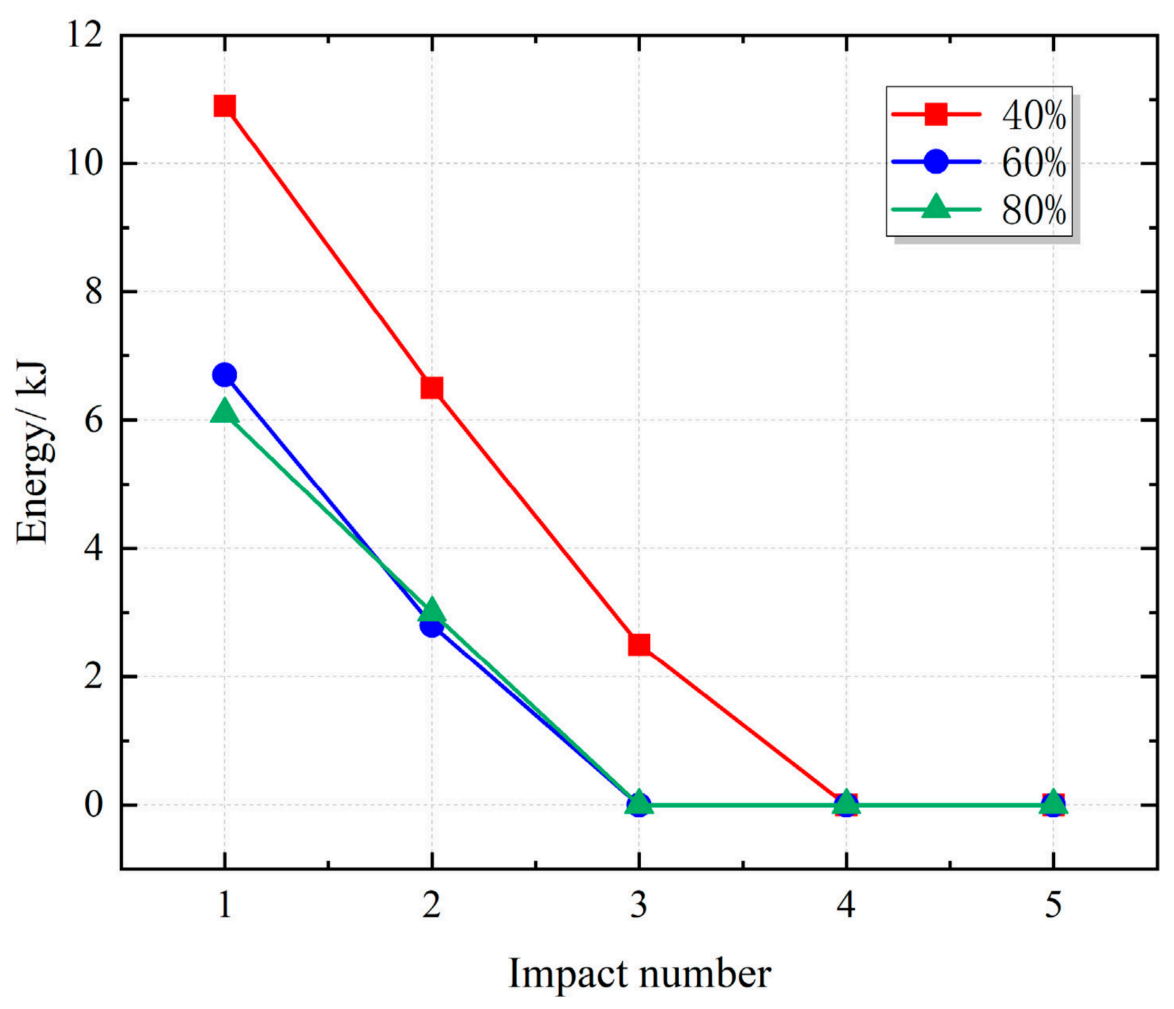
Typical energy change diagram of BFRP specimen under repeated impact.

Figure 10 presents the rebound energy of BFRP specimens with different nominal fiber mass fractions over five successive impacts, each conducted at an incident energy of 30 J. The rebound energy generally decreases over the repeated-impact sequence, indicating a reduction in the recoverable portion of the incident energy as the impact response evolves. The differences among fiber-content groups suggest that nominal fiber content influences energy dissipation and the evolution of the contact response under repeated loading.

**Figure 11:**
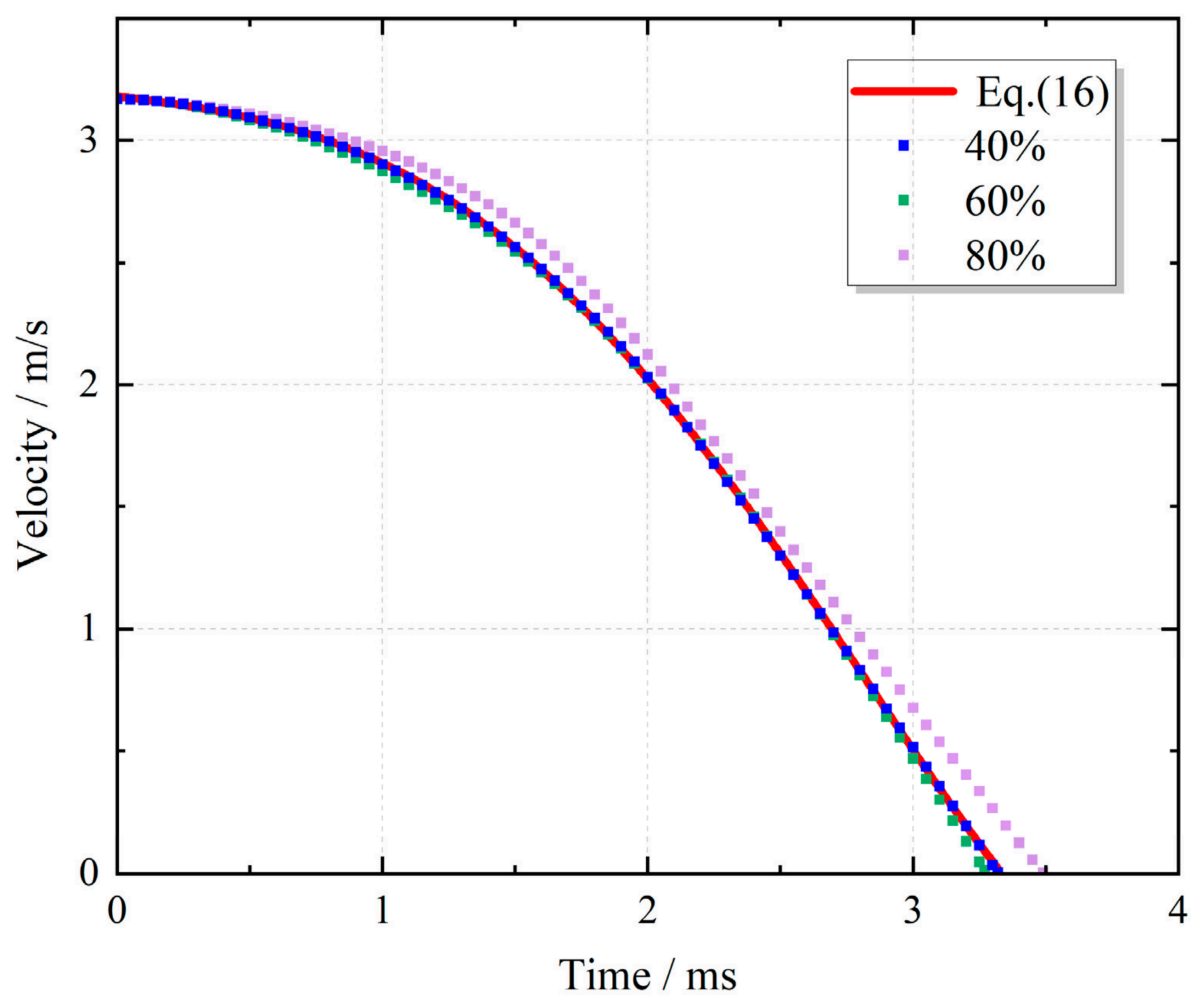
Punch velocity attenuation process of BFRP specimens with different nominal fiber contents under impact loading.

Figure 11 shows the variation in impactor velocity during penetration for BFRP specimens with different nominal fiber contents, together with the predictions obtained using Equation (16). The curves show a similar initial velocity among groups followed by a decrease during impact. Differences in the rate of velocity decay suggest that laminate response and effective stiffness influence the transmission and dissipation of impact energy.

(16) $$y=3.182+-0.128*x+-0.047*x^{2}+-0.097*x^{3}+-0.0072*x^{4}+0.0054*x^{5}$$

### 4.2. Model Validation and Parameter Analysis

**Figure 12:**
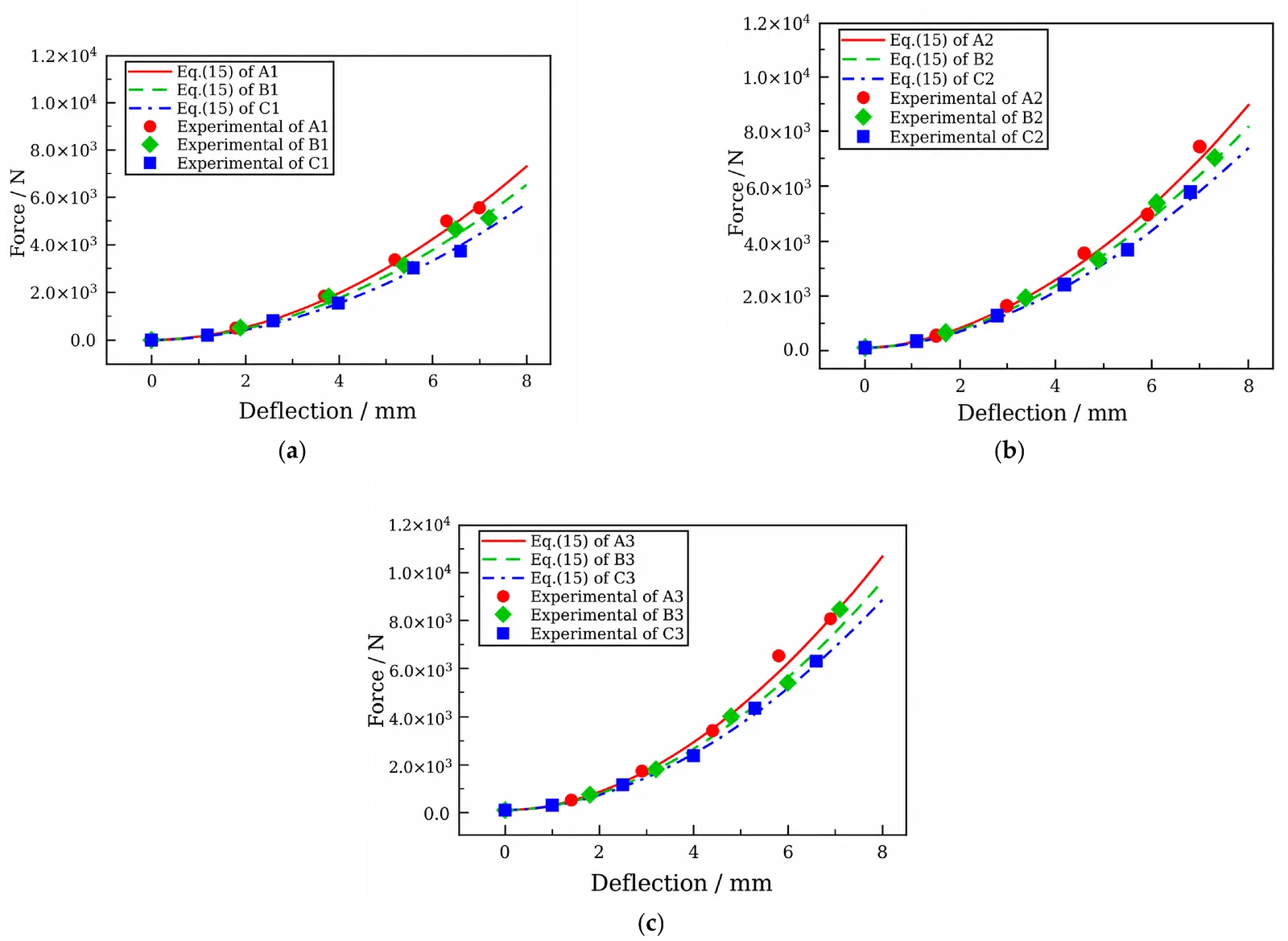
Predicted and experimental force–deflection responses of BFRP laminates with different fiber mass fractions and stacking sequences: (**a**) [0/90]; (**b**) [0/45/90/−45]; and (**c**) [±30].

Figure 12 compares the predicted and experimental force–deflection responses of all nine BFRP specimen configurations, A1–A3, B1–B3, and C1–C3. The specimen configurations and test conditions, including impact energy, nominal fiber mass fraction, stacking sequence, and specimen dimensions, are summarized in Table 3. The material and analytical parameters are described in Section 3.2. The three stacking-sequence subfigures provide a consistent basis for comparing the effects of fiber content and ply orientation on the measured and predicted force responses. The model captures the overall response trends under the investigated conditions, although its predictive accuracy varies among different specimen configurations.

Among the parameters involved in the model, *A*_d_ denotes the projected contact area of the impactor on the laminate and is determined by the geometry of the impactor. The parameters *σ*_c_ and *ρ*_t_ represent intrinsic material properties of the laminate. In contrast, the stiffness-related parameters are more sensitive to material composition and manufacturing conditions. In particular, variations in the ratio of the fiber elastic modulus *E*_f_ to the matrix elastic modulus *E*_m_ directly affect the equivalent stiffness of the BFRP laminate. The stacking sequence may also influence the effective elastic modulus *E*_p_ associated with the interlaminar region.

A comparison of the different model configurations shows that the investigated parameters affect the predicted response in different ways. At a fixed fiber content, increasing the fiber-to-matrix elastic modulus ratio leads to a higher predicted contact force owing to the increased equivalent bending stiffness of the laminate. In contrast, at a fixed elastic modulus ratio, variations in fiber content affect both the magnitude and evolution of the predicted response. Within the investigated parameter range, the response is more sensitive to stiffness-related parameters than to the interlaminar energy-dissipation parameter.

**Figure 13:**
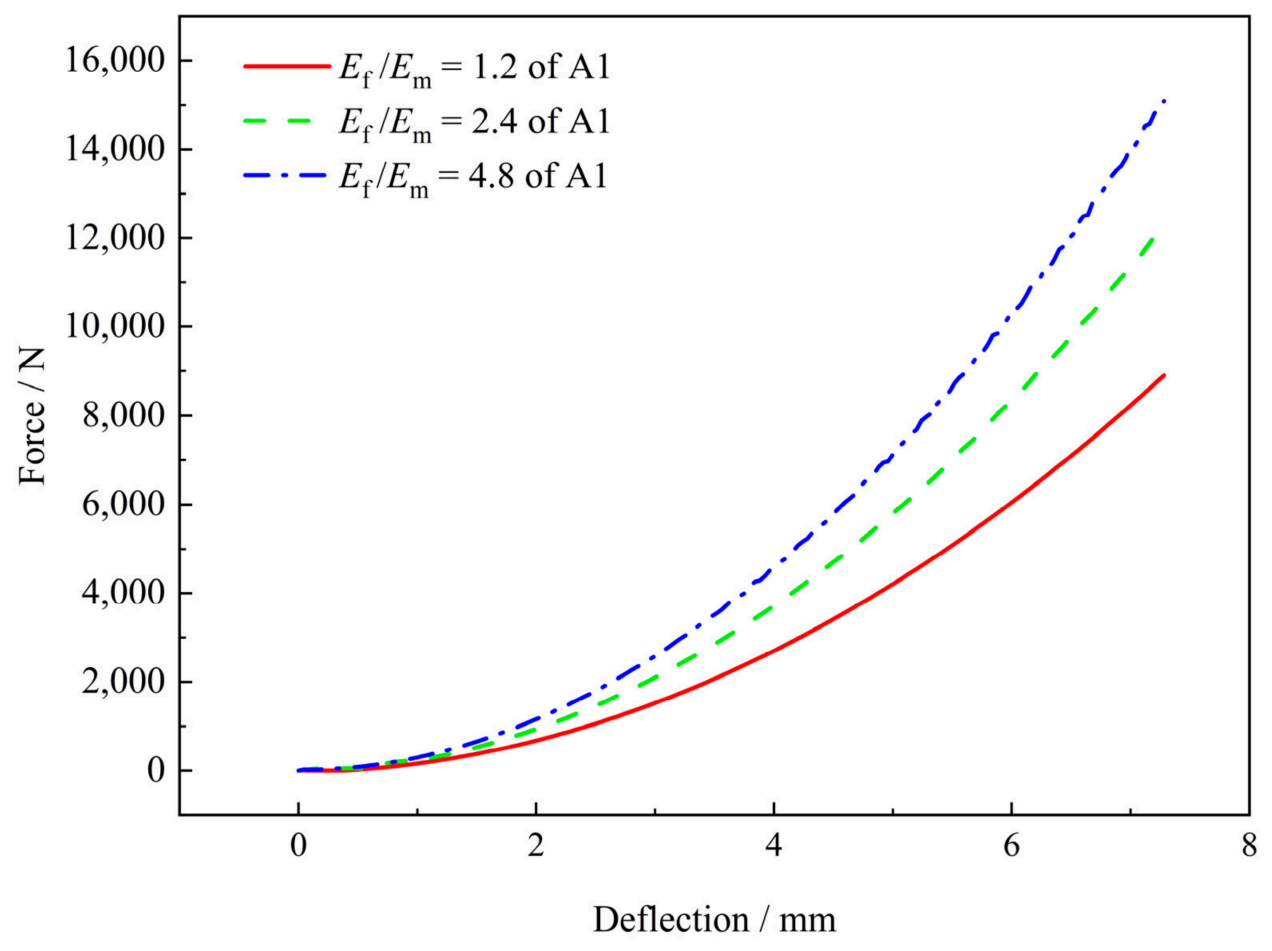
Effect of different values of *E*_f_/*E*_m_ on the theoretical model.

**Figure 14:**
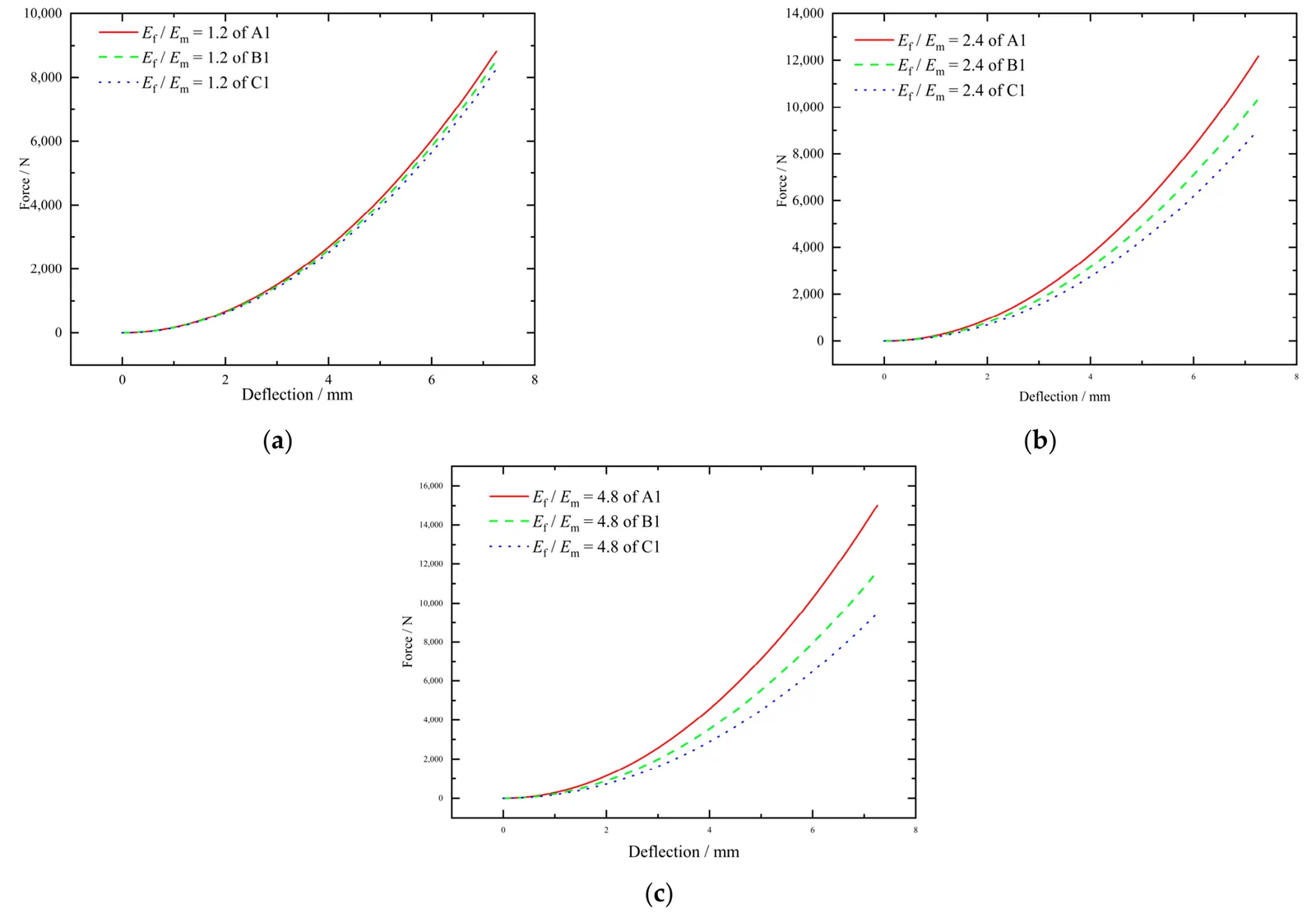
Predicted force–deflection responses of BFRP laminates with different fiber mass fractions at different fiber-to-matrix elastic modulus ratios: (**a**) *E*_f_/*E*_m_ = 1.2; (**b**) *E*_f_/*E*_m_ = 2.4; and (**c**) *E*_f_/*E*_m_ = 4.8.

This section further analyzes the model parameters based on the validated theoretical model. Since the effect of the ply laying method on the elastic modulus *E*_p_ of the delamination interface was not measured in this test, only the fiber content is analyzed. Figure 13 shows the difference in theoretical models under different *E*_f_*/E*_m_ for the A1 specimen with the same fiber content. As shown in Figure 13, with the increase in *E*_f_*/E*_m_, the peak contact force of the predicted curve gradually increases. This is because the increase in *E*_f_ directly increases the initial equivalent bending stiffness of the laminate, resulting in an increase in the bending deformation energy *E*_s_ corresponding to the unit deflection. Figure 14 shows the effect of different contents on the theoretical model under the same *E*_f_*/E*_m_ ratio. As shown in Figure 14, variations in fiber content noticeably affect the response results and impact resistance. With higher fiber content, the stiffness advantage of the fibers becomes more pronounced, but the proportion of the matrix decreases, which significantly increases the energy dissipation due to matrix cracking and delamination. When the difference between *E*_f_ and *E*_m_ is large, the response is strongly influenced by fiber content, indicating that matrix fracture and delamination are the main failure modes of the laminate during the impact process.

**Figure 15:**
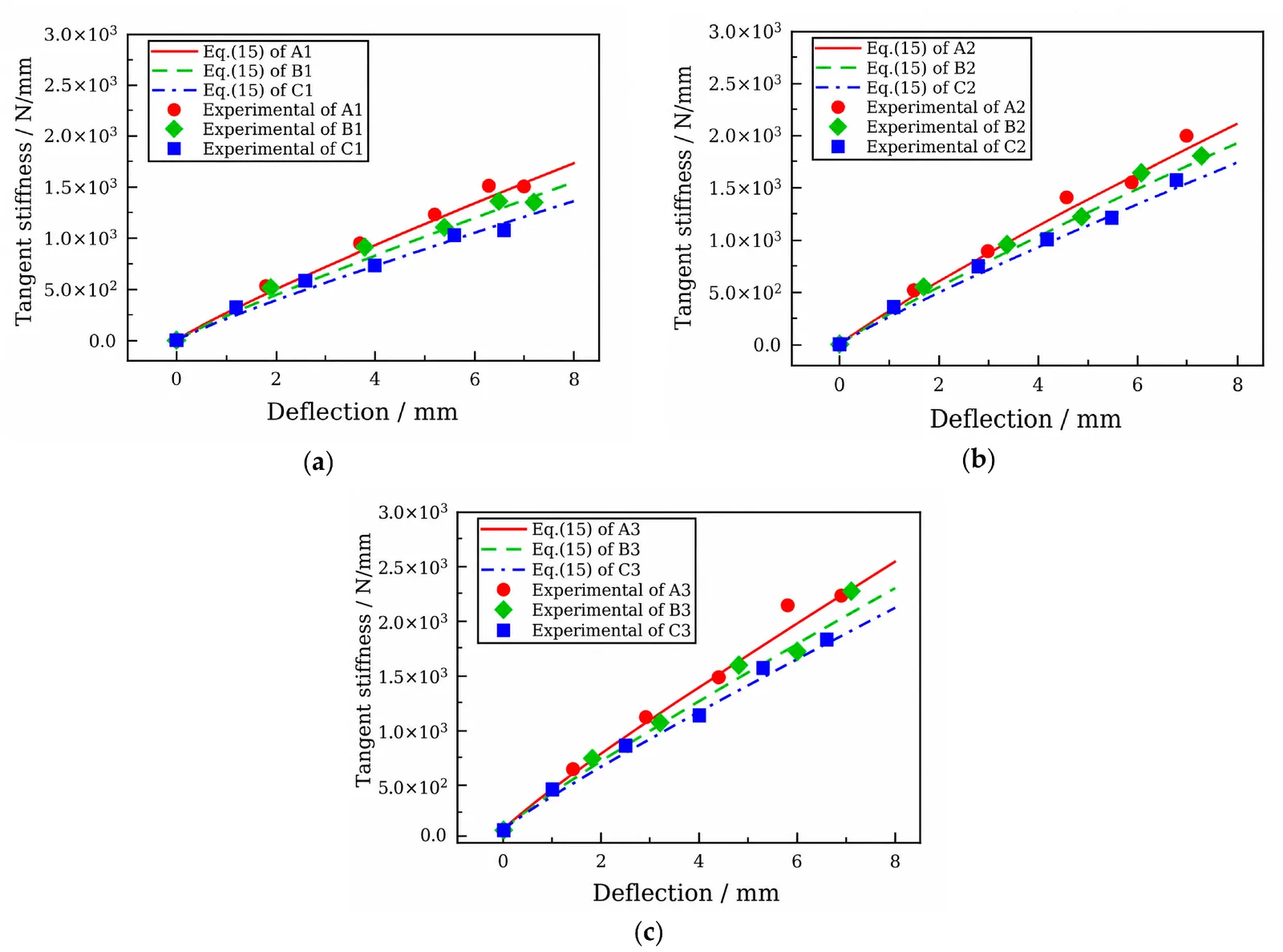
Tangent stiffness–deflection responses of BFRP laminates with different fiber mass fractions and stacking sequences: (**a**) [0/90]; (**b**) [0/45/90/−45]; and (**c**) [±30].

Figure 15 presents the tangent stiffness–deflection responses for the same nine specimen configurations shown in Figure 12. Tangent stiffness is defined as the local slope of the corresponding contact force–deflection curve at a given deflection. The theoretical and experimental tangent stiffness values were calculated from the predicted and measured force–deflection curves, respectively, and are expressed in N/mm. The predicted and experimental responses exhibit generally similar trends over the investigated configurations. The variations in tangent stiffness with deflection also differ among fiber contents and stacking sequences, consistent with the differences observed in the force–deflection responses.

The present results are generally consistent with earlier studies on the low-velocity impact behavior of fiber-reinforced composite laminates. Previous investigations have shown that stacking sequence influences contact response and damage tolerance [10,12,17]. Similarly, the [±30] laminates exhibited comparatively less severe surface damage, whereas the [0/90] laminates showed more pronounced damage, with the [0/45/90/−45] laminates displaying an intermediate response. The observed post-peak response fluctuations are consistent with previously proposed energy-dissipation mechanisms involving matrix cracking, delamination, and fiber failure [3,4,5,9], although the individual internal mechanisms were not independently quantified in this study. Compared with detailed numerical simulations, the proposed energy-based analytical model provides a simplified description of the macroscopic force–deflection and stiffness responses.

## 5. Conclusions

This study investigated the impact-penetration behavior of BFRP laminates with different fiber contents and stacking sequences. An energy-based impact-force model was developed to describe the impact response of the laminates under the investigated conditions. The main conclusions are as follows:

1. The proposed model reasonably describes the contact force–penetration depth relationship by accounting for bending deformation, interlaminar delamination, material failure, stiffness evolution, and penetration resistance. Within the investigated range of fiber contents and impact conditions, the predicted responses show reasonable agreement with the corresponding experimental results.
2. Fiber content influences both the impact response and damage characteristics of BFRP laminates. As the nominal fiber mass fraction increased from 40% to 80%, the post-peak response became increasingly oscillatory, consistent with progressive impact-induced damage. In contrast, specimens with lower fiber content exhibited smoother responses and less severe observed surface damage. Internal delamination and fiber failure are possible contributing mechanisms, but they were not independently quantified.
3. The stacking sequence also affects the impact performance of BFRP laminates. Among the investigated configurations, the [±30] angle-ply laminates exhibited the smallest observed damage area and a comparatively favorable impact response, whereas the [0/90] cross-ply laminates showed more severe damage under the tested conditions.
4. Stiffness evolution has an important influence on the predicted post-peak impact response. Within the investigated parameter range, the predicted contact response is more sensitive to stiffness-related parameters than to the interlaminar delamination energy parameter.

## Figures and Tables

**Table 1:** Mechanical properties of the BWR300-100 basalt fiber fabric and consolidated BFRP laminates.

Test Contents	Test Method	Tensile Breaking Strength N/mm	Intensity *σ*/MPa	Coefficient of Variation/%
Fabric performance warp direction	GB/T7689.5-2001	1920	/	3.7
Fabric performance weft	GB/T7689.5-2001	1959	/	2.4
Laminated slab performance bending	GB/T1449-2005	/	372	2.8
Performance compression of laminates	ISO 14126:1999	/	220	1.9

**Table 2:** Mean specimen thicknesses of the BFRP laminates (mm).

Group	Specimen 1 (mm)	Specimen 2 (mm)	Specimen 3 (mm)
A1	7.98	8.02	8.01
A2	8.03	7.99	8.02
A3	7.97	8.01	7.99
B1	8.04	8.00	7.98
B2	8.02	7.97	8.01
B3	7.99	8.03	8.02
C1	7.96	8.01	7.98
C2	8.02	7.99	8.04
C3	8.01	7.97	8.03

**Table 3:** Impact test conditions.

Specimens	Specimen Size mm × mm	Layer	Fiber Mass Fraction/%	Impact Energy/J
A1	100 × 100	[0/90]	40%	30
A2	100 × 100	[0/45/90/−45]	40%	30
A3	100 × 100	[±30]	40%	30
B1	100 × 100	[0/90]	60%	30
B2	100 × 100	[0/45/90/−45]	60%	30
B3	100 × 100	[±30]	60%	30
C1	100 × 100	[0/90]	80%	30
C2	100 × 100	[0/45/90/−45]	80%	30
C3	100 × 100	[±30]	80%	30

## Data Availability

The original contributions presented in the study are included in the article, further inquiries can be directed to the corresponding author.
